# Associations Between Neighborhood-Level Racial Residential Segregation, Socioeconomic Factors, and Life Expectancy in the US

**DOI:** 10.1001/jamahealthforum.2023.1805

**Published:** 2023-07-14

**Authors:** Sadiya S. Khan, Cyanna McGowan, Laura E. Seegmiller, Kiarri N. Kershaw

**Affiliations:** 1 Division of Cardiology, Department of Medicine, Northwestern University Feinberg School of Medicine, Chicago, Illinois; 2 Department of Preventive Medicine, Northwestern University Feinberg School of Medicine, Chicago, Illinois

## Abstract

This cross-sectional study evaluates the association between neighborhood-level residential segregation and life expectancy and aims to determine the proportion mediated by representative neighborhood-level socioeconomic factors.

## Introduction

Considerable disparities exist for life expectancy between non-Hispanic Black and White adults with root causes attributable to structural racism. Specifically, residential segregation at the neighborhood level has been associated with adverse health outcomes, which has largely been studied at the state^1^ and county levels.^2^

However, the effect of residential segregation perpetuated by discriminatory housing practices and related socioeconomic disadvantage requires analysis at the neighborhood level. Therefore, we sought to evaluate the association between neighborhood-level residential segregation and life expectancy and determine the proportion mediated by representative neighborhood-level socioeconomic factors.

## Methods

We obtained data on life expectancy at birth for 2010 to 2015 from the US Small-Area Life Expectancy Estimates Project^3^ and sociodemographic factors based on self-report from the American Community Survey for 2010 to 2014 (percentage of Black respondents, those without a bachelor’s degree, those living below the federal poverty level, and percentage unemployed). Residential segregation was calculated with the local Getis-Ord G_i_*statistic.^4^ The G_i_* statistic represents a *z* score, which indicates the difference in mean racial composition (percentage of Black individuals) in the census tract compared with the core-based statistical area (population of ≥10 000). More positive *z* scores reflect higher racial segregation. Segregation levels were modeled continuously and categorically: high (G_i_*, >1.96), medium (G_i_*, 0-1.96), and low (G_i_*, ≤0).^5^ The Northwestern University institutional review board determined this study to be exempt from review and informed consent due to the use of deidentified data. This study followed the STROBE reporting guideline.

We assessed the associations between residential segregation, socioeconomic factors, and life expectancy using a parallel multiple mediation analysis (Figure).^6^ Structural equation modeling was used to estimate the total effect of segregation on life expectancy and disaggregate the indirect effect of the socioeconomic factors (mediators assessed jointly in a single model) and the effect independent of the mediators and confounder.^6^ We also performed a sensitivity analysis limiting data to those census tracts with standard error of 3 or fewer years. All analyses were conducted in SAS, version 9.4 (SAS Institute). A 2-tailed *P* < .05 was considered statistically significant.

**Figure.**
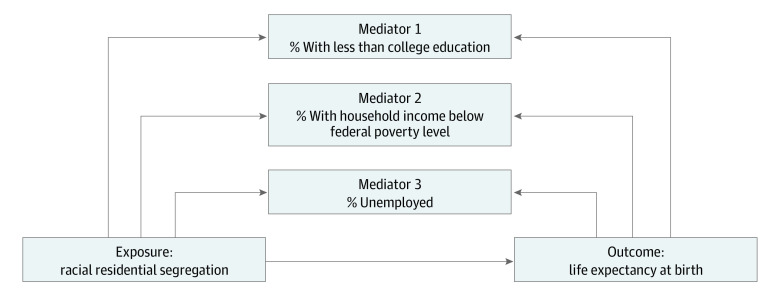
Proposed Pathway Analysis Model of Segregation and Life Expectancy Mediating pathways via census tract–level socioeconomic factors representing education, wealth, and employment in the association between racial residential segregation and life expectancy at birth. Structural equation modeling was applied to estimate the total effect of residential segregation (exposure) on life expectancy (outcome) and disaggregate the indirect effect of the socioeconomic factors (mediators assessed jointly in a single model) and the effect independent of the mediators and cofounders.

## Results

Among 63 694 included census tracts, mean (SD) life expectancy was 78.3 (4.0) years and was statistically significantly lower in neighborhoods with higher racial segregation (high, 75.1 [4.2] years; medium, 77.3 [3.8] years; and low, 79.3 [3.6] years). In high-segregated compared with low segregated neighborhoods, a higher mean (SD) percentage of residents lacked a college education (81% [13%] vs 69% [19%]), were living below the federal poverty level (24% [13%] vs 11% [8%]), and were unemployed (16% [8%] vs 8% [4%]). The association between racial segregation and life expectancy was statistically significantly mediated (β [SE]) by education (−0.201 [0.003]), poverty (−0.270 [0.005]), and unemployment (−0.052 [0.004]) (Table); the association between residential segregation and life expectancy independent of the 3 included socioeconomic mediators was also statistically significant (−0.276 [0.007] years). Similar findings were observed in the sensitivity analysis (Table).

**Table.** Associations Between Racial Residential Segregation, Census Tract–Level Socioeconomic Factors (Education, Wealth, and Employment), and Life Expectancy at Birth

Variable	β (SE)			% Mediated^b^
	Exposure mediator	Mediator outcome	Mediated indirect effect^a^	
**Complete analytic sample**				
Education	0.144 (0.002)	−1.401 (0.014)	−0.201 (0.003)	25
Poverty	0.217 (0.002)	−1.241 (0.016)	−0.270 (0.005)	34
Employment	0.218 (0.002)	−0.239 (0.016)	−0.052 (0.004)	7
Joint effect	NA	NA	−0.524 (0.006)	NA
**Sensitivity analysis in restricted analytic sample with SE ≤3 y**				
Education	0.142 (0.002)	−1.417 (0.014)	−0.200 (0.003)	25
Poverty	0.217 (0.002)	−1.261 (0.016)	−0.273 (0.005)	34
Employment	0.215 (0.002)	−0.207 (0.016)	−0.045 (0.004)	6
Joint effect	NA	NA	−0.518 (0.006)	NA

Abbreviation: NA, not applicable.

^a^ The indirect effect represents the association between the exposure and the outcome through the mediators.

^b^ Represents the indirect effect for each mediator/total effect.

## Discussion

This nationwide cross-sectional study demonstrated that residing in a highly segregated neighborhood was associated with statistically significantly lower life expectancy by 4 years, which was partially mediated by neighborhood-level socioeconomic factors. These findings help to quantify the contribution of residential segregation as a key structural driver of racial inequities. This study uses a more direct measure of racial residential segregation (G_i_* coefficient) instead of a proxy relying only on the percentage of Black residents in a census tract, which does not account for the demographic composition of the surrounding area. Although we selected 3 key mediators, other factors related to structural racism also likely mediate the association between segregation and life expectancy, including access to health care, housing stability, and environmental pollution. Limitations of this analysis include the ecologic study design that cannot confer causation and the potential for biasing the measured effects due to omitted variables in the structural equation modeling. These findings contribute to growing evidence of place-based disadvantage due to residential segregation that limit access to health-promoting factors (eg, education, employment, wealth) and may enhance exposure to health-harming factors (eg, air pollution) in contemporary estimates of life expectancy.

## Notes

Supplement. Data Sharing Statement
