# Supplementary material for: Associations Between Neighborhood-Level Racial Residential Segregation, Socioeconomic Factors, and Life Expectancy in the US
Source: JAMA Health Forum. 2023 Jul 14;4(7):e231805. doi: 10.1001/jamahealthforum.2023.1805 (PMC10349335; doi:10.1001/jamahealthforum.2023.1805)
Supplement: Supplement. — Data Sharing Statement [file jamahealthforum-e231805-s001.pdf]

Data Sharing Statement

Khan. Associations Between Neighborhood-Level Racial Residential Segregation, Socioeconomic Factors, and Life Expectancy in the US. *JAMA Health Forum*. Published July 14, 2023. doi:10.1001/jamahealthforum.2023.1805

Data
Data available: Yes
Data types: Deidentified participant data
How to access data: These data are publicly available: https://www.cdc.gov/nchs/nvss/usaleep/usaleep.html
When available: With publication

Supporting Documents
Document types: None

Additional Information
Who can access the data: These data are publicly available: https://www.cdc.gov/nchs/nvss/usaleep/usaleep.html
Types of analyses: These data are publicly available: https://www.cdc.gov/nchs/nvss/usaleep/usaleep.html
Mechanisms of data availability: These data are publicly available: https://www.cdc.gov/nchs/nvss/usaleep/usaleep.html
Any additional restrictions: None
